# Cr incorporated phase transformation in Y_2_O_3_ under ion irradiation

**DOI:** 10.1038/srep40148

**Published:** 2017-01-16

**Authors:** N. Li, S. K. Yadav, Y. Xu, J. A. Aguiar, J. K. Baldwin, Y. Q. Wang, H. M. Luo, A. Misra, B. P. Uberuaga

**Affiliations:** 1 Materials Physics and Applications Division, MPA-CINT, Los Alamos National Laboratory, Los Alamos, New Mexico 87545, USA; 2 Materials Science and Technology Division, MST-8, Los Alamos National Laboratory, Los Alamos, New Mexico 87545, USA; 3 Department of Chemical and Materials Engineering, New Mexico State University, Las Cruces, New Mexico 88003, USA; 4 Idaho National Laboratory, Idaho Falls, Idaho, 83415, USA; 5 Department of Materials Science and Engineering, University of Michigan, Ann Arbor, Michigan 48109, USA

## Abstract

Under irradiation, chemical species can redistribute in ways not expected from equilibrium behavior. In oxide-dispersed ferritic alloys, the phenomenon of irradiation-induced Cr redistribution at the metal/oxide interfaces has drawn recent attention. Here, the thermal and irradiation stability of the FeCr/Y_2_O_3_ interface has been systematically studied. Trilayer thin films of 90 nm Fe - 20 at.% Cr (1^st^ layer)/100 nm Y_2_O_3_ (2^nd^ layer)/135 nm Fe - 20 at.% Cr (3^rd^ layer) were deposited on MgO substrates at 500 °C. After irradiation, Cr diffuses towards and enriches the FeCr/Y_2_O_3_ interface. Further, correlated with Cr redistributed into the oxide, an amorphous layer is generated at the interface. In the Y_2_O_3_ layer, the original cubic phase is observed to transform to the monoclinic phase after irradiation. Meanwhile, nanosized voids, with relatively larger size at interfaces, are also observed in the oxide layer. First-principles calculations reveal that Cr substitution of Y interstitials in Y_2_O_3_ containing excess Y interstitials is favored and the irradiation-induced monoclinic phase enhances this process. Our findings provide new insights that may aid in the development of irradiation resistant oxide-dispersed ferritic alloys.

By virtue of a high density of metal/oxide (M/O) interfaces, oxide dispersion strengthened (ODS) steels (Fe-Cr alloys containing a dispersion of nanosized oxide particles) present unprecedented radiation tolerance and have been regarded as one of the most promising material candidates for cladding and structural components in future advanced reactors^1^,^2^,^3^. Extensive studies have uncovered their extraordinary mechanical properties, including remarkable thermal stability up to 1000 °C^4^,^5^,^6^; high tensile, creep and fracture strengths over a wide range of temperatures^7^,^8^,^9^,^10^,^11^,^12^,^13^,^14^; and resistance to helium embrittlement^15^,^16^,^17^,^18^,^19^. Interior nano-sized oxides present a strong pinning effect to maintain the high density of dislocations and to restrain grain growth at high operating temperatures^20^. However, despite these advantages, the ODS steels themselves are not immune to irradiation: under irradiation, solute atoms redistribute at the M/O interface, which results in the degradation of the existing oxide phases^21^,^22^,^23^ and/or the nucleation of deleterious phases. For example, under irradiation, recoil resolution happens in one type of ODS ferritic steels (Fe − 13Cr − 1.5Mo + 1TiO_2_ + 0.5Y_2_O_3_)^24^ and halos of finer particles have been observed^25^. When the radiation dose reaches 60 dpa at approximately 500 °C, the finest-scale oxides display a tendency to disappear^26^,^27^. Finally, radiation induced Cr segregation causes the precipitation of α′, which is regarded as the major reason for mechanical hardening and embrittlement^1^,^10^,^28^,^29^,^30^.

Y_2_O_3_ powders, as one of the major components in ODS steels, have been added together with FeCr based powders (containing Ti, W, or Mo) during synthesis^31^,^32^. Thus, the study of Y_2_O_3_ phase stability and radiation response is of particular relevance to ODS and has attracted abundant attention recently. Under irradiation, Y_2_O_3_ phase stability is closely related to the local stress state. *In situ* Kr ion irradiation studies induced an evident change from pristine amorphous to a monoclinic structure when Y_2_O_3_ was constrained by surrounding Fe layers^33^. On the other hand, when Y_2_O_3_ is irradiated as free-standing crystalline powders, a structural transformation from the cubic phase to a quasi-amorphous phase and finally to a monoclinic phase has been uncovered under swift Xe ions irradiation^34^,^35^. This is mainly due to the accumulation of internal stress, which results from irradiation-induced defects such as prismatic dislocation loops^34^,^36^. Similar cubic-to-monoclinic phase transformation was also observed in other bixbyite structured sesquioxides under irradiation^37^. However, in these studies, either there was no metallic phase or the metallic phase was chemically pure Fe. In real ODS steels, which contain a large number of alloying elements, radiation induced chemical redistribution will occur at the metal/Y_2_O_3_ interface, which may significantly influence the evolution of the oxide phase. For example, our previous studies uncovered that the irradiation-induced incorporation of Cr in TiO_2_ enhances the tendency of amorphization^38^ and, in MgO, causes the formation of MgCr_2_O_4_ spinel^39^.

Here, layer-structured FeCr/Y_2_O_3_ thin films are grown via the magnetron sputtering technique, in which the M/O interface structure and crystallography have been characterized before and after irradiation. Under Ni ion irradiation at 500 °C, we observed the segregation of Cr towards the FeCr/Y_2_O_3_ interface and a phase transformation in Y_2_O_3_ from the original cubic phase to the monoclinic phase. Upon incorporation of Cr, the oxide layer tends to become amorphous. In addition, first-principles calculations show that it is thermodynamically favorable to substitute Y with Cr in Y_2_O_3_ containing Y interstitials.

## Results

**Figure 1:**
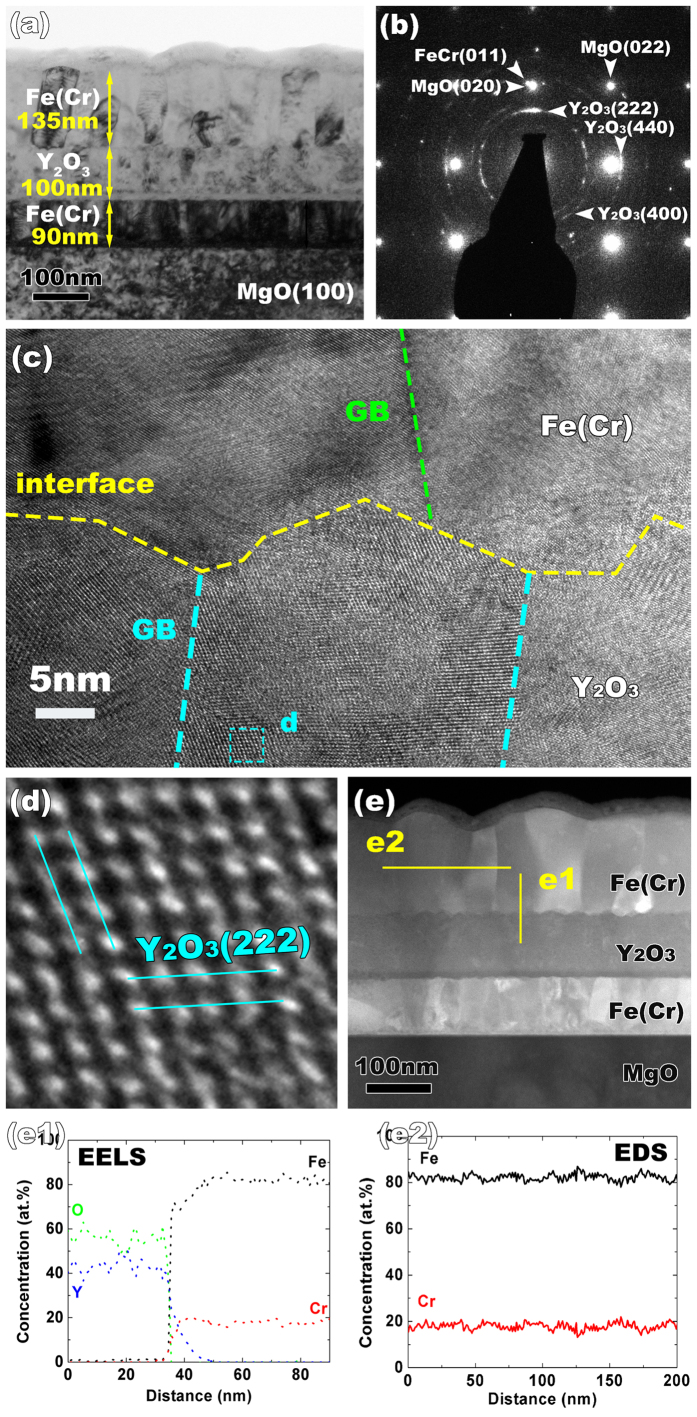
(**a**) Bright-field cross-sectional TEM image of pristine the FeCr/Y_2_O_3_ trilayer thin film. (**b**) is the selected area electron diffraction pattern. (**c**) HRTEM micrograph of the Y_2_O_3_/FeCr interface. (**d**) Magnified HRTEM image of cubic Y_2_O_3_ indicated by the blue dashed square in (**c**). (**e**) Dark field STEM image reveals a chemically abrupt interface. (e1) EELS line scan across the M/O interface and (e2) EDS composition profile across the grain boundary in FeCr layer.

The microstructure of the sputtered FeCr/Y_2_O_3_ trilayer thin film is presented in Fig. 1a. The individual layer thickness for the first FeCr, the second Y_2_O_3_ and the third FeCr layer are 90, 100, and 135 nm, respectively. The corresponding diffraction pattern (DP), as presented in Fig. 1b, indicates the cubic phase (space group: Ia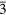) of Y_2_O_3_ and weak Y_2_O_3_ (222) texture. Upon the beginning of depositing the Y_2_O_3_ on the first FeCr layer, probably due to the slower diffusion rate of sputtered Y atoms compared to O atoms, more oxygen arrive at the surface of the first FeCr layer, resulting in the formation of a thin oxygen-rich amorphous layer at the bottom FeCr/Y_2_O_3_ interface. Thus, in this work, we will focus on the evolution of the upper interface, as it is sharp. The upper interface (2^nd^ Y_2_O_3_/3^rd^ FeCr) structure has been explored using high resolution transmission electron microscopy (HRTEM). As shown in Fig. 1c, the interface is faceted. The grain size in Y_2_O_3_ is small, about 20 nm on average. Figure 1d is the magnified HRTEM image of Y_2_O_3_, from the area indicated by the blue dashed square in Fig. 1c, and this image confirms the cubic structure of Y_2_O_3_ as found in the diffraction pattern. Figure 1e shows a dark field scanning TEM (STEM) image of the pristine sample with Y_2_O_3_ in dark contrast. The corresponding chemical composition was analyzed by EELS (in Fig. 1e1) and energy dispersive X-ray spectroscopy (EDS) (in Fig. 1e2), with the direction of the electron beam parallel to the interface. Across the interface (in Fig. 1e1), the chemical profiles of elements Fe and O are sharp. In comparison, Y seems to extend a little into the FeCr layer. The subtle deviation between the Y and O chemical profiles may contribute to the difference of the sputtered ions’ mobility towards the substrate during the deposition process. In the oxide layer, the chemical concentration of Y is slightly higher than 40 at.%. In addition, EDS analysis has been performed in the FeCr layer across several grain boundaries (in Fig. 1e2) and the as-deposited Cr profile presents very little variation near the boundaries. Thus, the extent of Cr segregation to grain boundaries in the pristine sample is negligible.

**Figure 2:**
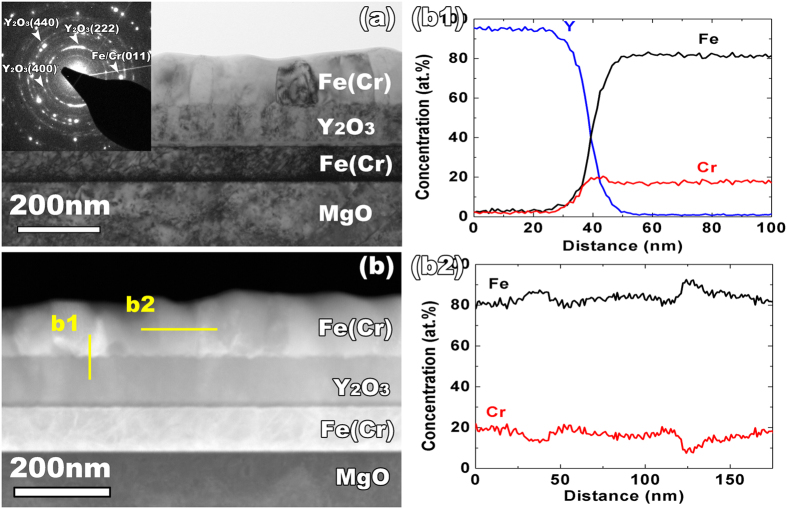
(**a**) Cross-sectional TEM micrograph and DP show no obvious change of microstructure of the FeCr/Y_2_O_3_ trilayer thin film after annealing. (**b**) Corresponding dark field STEM image with the position of the EDS line scan. (b1) and (b2) are EDS composition profiles across the M/O interface and grain boundaries in FeCr layer. The diffusion of Cr towards the M/O interface is negligible, in compared to the Cr depletion at grain boundaries in FeCr layer.

As a base line for comparison with the irradiated sample, the trilayer sample was annealed for one hour at 500 °C, the same time and temperature used for the irradiation. As shown in Fig. 2a and b, after annealing, there is no clear structural change in either the FeCr or Y_2_O_3_ layers. The DP in Fig. 2a reveals no formation of any new phases after annealing. By comparing the chemical profiles of Cr before and after annealing (Fig. 1e1 vs. Fig. 2b1), the tendency of Cr to diffuse towards the interface is negligible. This is different from our previous FeCr/TiO_2−x_ studies, in which Cr diffused into the TiO_2−x_ layer during thermal annealing^38^. The EDS scan across the grain boundary in the FeCr layer uncovers a limited extent of Cr depletion at the boundaries (in Fig. 2b2).

**Figure 3:**
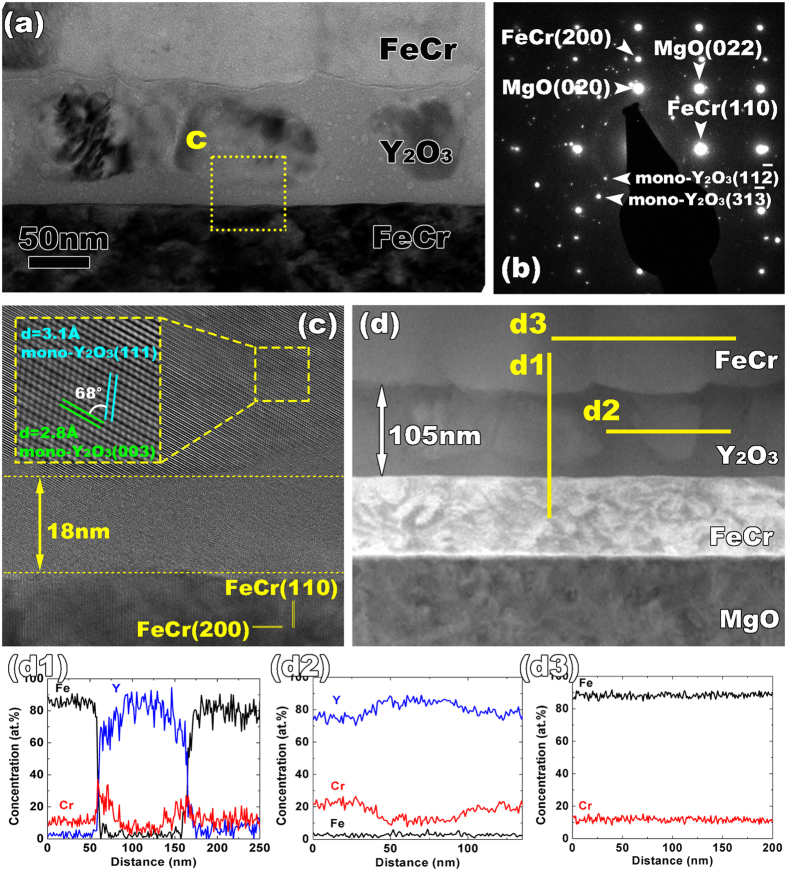
(**a**) TEM image of the trilayer structure after irradiation with 10 MeV Ni ions. The yellow dashed box indicates the region that is shown in higher magnification in (**c**). (**b**) The corresponding diffraction pattern uncovers an irradiation-induced monoclinic phase. (**c**) An amorphous layer with a thickness of 18 nm is generated at the M/O interface after irradiation. The pristine cubic Y_2_O_3_ transforms to the monoclinic phase. (**d**) Dark field STEM image of irradiated multilayers. (d1–d3) EDS chemical analyses at different regions indicate the situation of Cr diffusion at different boundaries.

The sample was irradiated by 10 MeV Ni^3+^ ions at 500 °C to a fluence of 10^16^ ions/cm^2^. Based on SRIM calculations^40^, the average dpa is around 10 in the metal layers and around 4 in the oxide layer. The displacement threshold energies used for all 4 species were 25 eV while the calculations were performed in the “quick” Kinchin and Pease mode. After irradiation, while the layered structure is sustained and the two FeCr layers maintain their crystallinity, Fig. 3a reveals that there are significant changes to the morphology of the Y_2_O_3_ layer. A newly generated amorphous layer is detected at the upper interface after irradiation, resulting in the Y_2_O_3_ layer being comprised of both crystalline and amorphous domains. The average crystalline grain size is around 60 nm, which is larger than the pristine sample (around 20 nm). These grains reside in the center of the Y_2_O_3_ layer and are surrounded by amorphous material both near the FeCr/Y_2_O_3_ interfaces (Fig. 3c) but also between them within the Y_2_O_3_ layer. Concurrent with the amorphization, voids are formed in the Y_2_O_3_ layer (as displayed in Fig. 3a via the lighter contrast spots), with relatively larger sizes at interfaces. The radiation-induced structural changes at the interface have been explored by the HRTEM in Fig. 3c. The cubic Y_2_O_3_ phase changes to the monoclinic phase after irradiation. The corresponding DP in Fig. 3b also reveals the formation of the monoclinic phase. In addition, the thickness of the oxide layer expands to 104 nm (from 100 nm, approximately 4% swelling) after irradiation, accommodating the generation of voids. STEM studies were also carried out on the ion-irradiated trilayer thin film. As shown in Fig. 3d, multiple EDS line scans across different boundaries have been performed. Figure 3d1 presents the profile of the chemical elements crossing both M/O interfaces (bottom-up direction) and clearly shows the segregation of Cr at both M/O interfaces after irradiation. In addition, the location of the irradiation-induced amorphous layer is correlated with Cr enrichment. In the Y_2_O_3_ layer, an EDS line scan is carried out parallel to the M/O interface, across the boundary of the crystalline and amorphous oxide phases (in Fig. 3d2). Similar to the interfacial regions, Cr enrichment in the amorphous region within the oxide layer has been observed, even in the center of the oxide layer. The third EDS scan is across the grain boundaries in the FeCr layer; no Cr segregation is seen at these boundaries (in Fig. 3d3). In fact, the Cr profile in Fig. 3d3 is even flatter than for the as-deposited and annealed cases (Fig. 1e2 and 2b2). However, the relative amount of Cr in the FeCr layer is smaller than it was in those cases, a reflection of the interdiffusion of Cr into the oxide layer.

## Discussion

Under irradiation, we observe multiple phase transformations in the Y_2_O_3_ layer, from an initial cubic structure to either amorphous or monoclinic phases. Further, the amorphization, both at the M/O interface and within the Y_2_O_3_ layer itself, occurs concurrently with Cr enrichment at the same locations. Previous work uncovered radiation-induced nucleation of the monoclinic phase in Y_2_O_3_^35^,^36^, however, the amorphization of Y_2_O_3_ due to ion irradiation has not been reported, to the best of our knowledge. Sickafus *et al*. systematically investigated the phase stability of various A_2_O_3_ cubic oxides under irradiation and found that generally they are very resistant to amorphization^37^,^41^,^42^. In contrast, we observe amorphization of Y_2_O_3_ after a dose of only 4 dpa. Our hypothesis is that incorporation of Cr into the oxide layer is one of the major reasons for accelerating this amorphization process. With Cr atoms incorporating into Y_2_O_3_, the bonding strength between metal and oxygen ions may be affected in a way that may induce more nucleation of point defects under irradiation. Meanwhile, the presence of impurity Cr atoms may diminish the re-crystallization efficiency of the damage cascade itself, dramatically enhancing the amorphization process^43^. On the other hand, phase stability of Y_2_O_3_ in irradiation environments depends strongly on the grain size of crystalline oxides: phase transformation to amorphous is easier at smaller grains^44^,^45^.

To probe our hypothesis that Cr drives the amorphization transformation, the mechanism of Cr incorporation into Y_2_O_3_ has been explored by density functional theory (DFT) calculations. Here we consider the situation of Fe and Cr as substitutional or interstitial dopants in stoichiometric Y_2_O_3_ and Y_2_O_3_ containing an yttrium interstitial, as would be expected from the irradiation, as discussed below. The formation energy of the substitutional reaction is calculated as


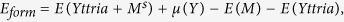


where 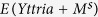 and 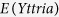 are the DFT energies of Y_2_O_3_ (stoichiometric and sub-stoichiometric) with and without metal M (Fe or Cr) substitution, respectively. *μ(Y*) is the chemical potential of Y and *E(M*) is the bulk DFT energy (or chemical potential) of metallic Fe or Cr. Taking the bulk value (cohesive energy) as the chemical potential for the metallic species is a reasonable assumption, as this is good representation of the FeCr thin film itself, the region where the metal originates in our experiments. Thus, this reaction represents taking a metal atom from the film and substituting it for Y within the oxide. The Y atom is subsequently placed in an arbitrary reference phase, which we will treat as a range of possible chemical potentials for Y. The formation energy of inserting the metal M (Fe or Cr) species into Y_2_O_3_ as an interstitial defect, without any substitution, is calculated as:


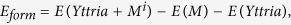


where 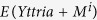 is the DFT energy of Y_2_O_3_ (stoichiometric and sub-stoichiometric) with a metal M (Fe or Cr) interstitial.

For all cases neutral defects are considered. For the cases in which we are simply changing the identity of one species (i.e. substituting Cr or Fe for Y), the charge state for both systems is the same and we expect that, in comparing the energies of the two systems, the value of that charge is relatively unimportant, as the key energetic factor, the typical qε_F_ term (where q is the net charge of the defect and ε_F_ is the Fermi level), exactly cancels in comparing those energies. We thus expect the neutral case to be representative of the difference in energy of these two systems. In the case in which the number of atoms does change, such as comparing Cr or Fe substitution for Y versus insertion as an interstitial, the charge state does matter. However, because the Fermi level of Fe and Cr is close to the conduction band of Y_2_O_3_^46^,^47^ the qε_F_ will be large (maximizing the energy of creating holes) and thus will dominate over any differences in the energetics associated with changes in charge state. Thus, in this case, the neutral case represents a lower bound on the relative energies of the defects, as we have confirmed for the monoclinic case, comparing the 3+ and neutral cases.

**Figure 4:**
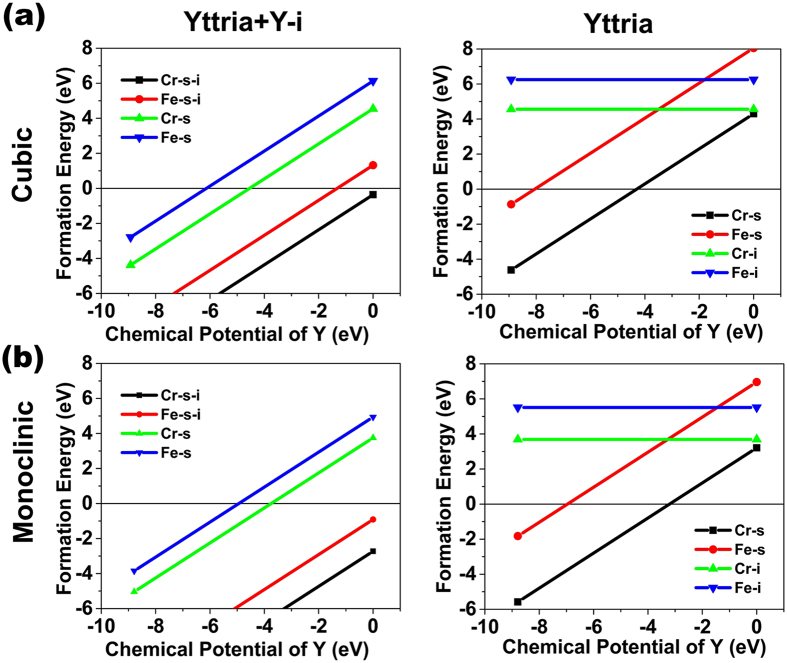
Defect formation energies as a function of Y chemical potentials in (**a**) cubic phase Y_2_O_3_ and (**b**) monoclinic phase Y_2_O_3_. Plots show the formation energies of dopant reactions as a function of Y chemical potentials: For Y_2_O_3_ containing a Y interstitial (Y_2_O_3_ Y-i) Fe and Cr substitution (Fe-s and Cr-s) at a native (bulk) Y site and Fe or Cr substitution (Fe-s-i or Cr-s-i) at the Y interstitial site were considered; Fe and Cr interstitials (Fe-i and Cr-i) and Fe and Cr substitution for bulk Y (Fe-s and Cr-s) were considered in stoichiometric Y_2_O_3_.

Figure 4 shows the defect formation energy as a function of the yttrium chemical potential, which, for us, is an unknown variable. The largest possible range for the chemical potential of yttrium is obtained from the stability limits of Y_2_O_3_ with respect to metallic yttrium and molecular oxygen (for more details refer to Ref. 47). Several positions for the substitutional defect is considered, particularly in the cases where there is an yttrium interstitial already within Y_2_O_3_; here we report only the most stable case. In comparing all of these scenarios, Cr substitution or interstitial formation is always preferred over the same reaction involving Fe. Further, Cr substitution is preferred over the direct formation of interstitials. In the case of substituting Y with Cr in Y_2_O_3_ containing Y interstitials, Cr significantly prefers to replace the Y interstitial as compared to a bulk Y. Finally, these trends are even greater for the monoclinic phase than for the cubic phase, suggesting that, once the irradiation induces the change in Y_2_O_3_ from cubic to monoclinic, the thermodynamic driving force for Cr incorporation and substitution into Y_2_O_3_ increases.

If Cr does indeed destabilize the material and accelerate amorphization, this will be more likely to occur in the monoclinic phase than in the cubic phase. The presence of voids in the oxide layer suggests that there are excess concentrations of Y interstitials within the material, further enhancing the substitution of Cr into the Y_2_O_3_ layer. Thus, both the irradiation-induced phase transformation of Y_2_O_3_ to the monoclinic phase and the reduced annihilation of Y interstitials (related to the formation of voids) lead to greater Cr substitution and destabilization of the oxide structure. From the perspective of thermodynamics, substitution of Y interstitials by Cr is exothermic, indicating there is a thermodynamic driving force for Cr incorporation into the oxide, the kinetics of which may be enhanced under irradiation because of the excess defect concentrations.

One other interesting observation from Fig. 3 is that the amorphization of the oxide occurs not only at the interface with FeCr, but also within the layers, between the remaining crystalline grains of Y_2_O_3_. Further, we observe enhanced Cr concentrations in these amorphous regions as well. This suggests that there is enhanced Cr diffusion along Y_2_O_3_ grain boundaries, allowing Cr to interdiffuse into the layer and enhance amorphization at the grain boundaries as well.

In summary, FeCr/Y_2_O_3_/FeCr trilayer thin films were subjected to either annealing at 500 °C for 1 hour or 10 MeV Ni^3+^ ion irradiation at 500 °C. The redistribution of Cr due to annealing is negligible. However, after ion irradiation, Cr preferentially diffuses towards and segregates at the FeCr/Y_2_O_3_ interface. Cr incorporation into the oxide layer, which can be induced by irradiation, enhances amorphization. Meanwhile, nanosized voids are formed in the Y_2_O_3_ layer, with relatively larger size at interfaces. First-principles calculations show that it is thermodynamically favorable to substitute Y with Cr in Y_2_O_3_ containing Y interstitials, which would likely be present due to the radiation damage. Diffusion of Cr into the oxide layer may influence the bond strength of cubic Y_2_O_3_ crystal and enhances amorphization under irradiation.

## Methods

### Experimental details

A trilayer thin film of Fe80 at.% Cr20 at.%/Y_2_O_3_/Fe 80 at.% Cr 20 at.% was deposited on a MgO (100) substrate at 500 °C. The deposition rate for FeCr and Y_2_O_3_ were controlled to be <5 Å/s. Ion irradiation experiments were performed at the Ion Beam Materials Laboratory at Los Alamos National Laboratory (LANL). 10 MeV Ni^3+^ ions were utilized to irradiate samples at 500 °C to a fluence of 10^16^ ions/cm^2^. The irradiation is performed at such high energy so that all Ni ions are implanted deep into the MgO substrate and not likely to modify the chemistry of the film. The elapsed time of the irradiation was about 1 hour. For comparison, a second as-synthesized sample was annealed at 500 °C for 1 hour. Both ion irradiation and annealing experiments have been performed under high vacuum conditions (~10^−8^ torr). TEM samples were prepared by mechanical polishing to a thickness of about 30 μm on a MultiPrep System and ion-milling to electron transparency on a Gatan PIPS. Analytical microscopy was performed on the image-corrected FEI Titan at LANL, operating in TEM mode at 300 keV equipped with a Gatan Tridiem electron energy loss image filter (GIF). Scanning TEM high angle annular dark field (HAADF) and electron energy loss (EEL) spectral imaging were performed using the probe-corrected FEI Titan and 100 kV Nion UltraSTEM at Oak Ridge National Laboratory.

### DFT simulations

Density functional theory (DFT) calculations, using the Vienna Ab initio Simulation Package (VASP)^48^, were performed, in which the Perdew, Burke, and Ernzerhof (PBE)^49^ generalized gradient approximation (GGA) exchange-correlation functional and the projector-augmented wave (PAW) method^50^ were employed. For all calculations, a plane wave cutoff of 500 eV for the plane wave expansion of the wave functions and a Monkhorst-Pack k-point grid of 2 × 2 × 2 for Brillouin zone integration were used to obtain highly accurate forces. Because of the magnetic structure of Cr and Fe, spin polarized calculations were considered for all cases. Force tolerance for the structural relaxation was 0.05 eV/Å. We use a cubic bixbyite structure with 80 atoms and a monoclinic crystal structure with 90 atoms to compute defect states.

## Additional Information

**How to cite this article**: Li, N. *et al*. Cr incorporated phase transformation in Y_2_O_3_ under ion irradiation. *Sci. Rep.* **7**, 40148; doi: 10.1038/srep40148 (2017).

**Publisher's note:** Springer Nature remains neutral with regard to jurisdictional claims in published maps and institutional affiliations.
